# Insights into an Immunotherapeutic Approach to Combat Multidrug Resistance in Hepatocellular Carcinoma

**DOI:** 10.3390/ph14070656

**Published:** 2021-07-09

**Authors:** Aswathy R. Devan, Ayana R. Kumar, Bhagyalakshmi Nair, Nikhil Ponnoor Anto, Amitha Muraleedharan, Bijo Mathew, Hoon Kim, Lekshmi R. Nath

**Affiliations:** 1 Department of Pharmacognosy, Amrita School of Pharmacy, Amrita Vishwa Vidyapeetham, AIMS Health Science Campus, Kochi 682041, Kerala, India; aswathyrd@aims.amrita.edu (A.R.D.); ayanark@aims.amrita.edu (A.R.K.); bhagyalakshmi@pharmacy.aims.amrita.edu (B.N.); 2 The Shraga Segal Department of Microbiology, Immunology, and Genetics, Faculty of Health Sciences, Ben-Gurion University of the Negev, P.O.B. 653, Beer Sheva 84105, Israel; antop@post.bgu.ac.il (N.P.A.); muraleed@post.bgu.ac.il (A.M.); 3 Department of Pharmaceutical Chemistry, Amrita School of Pharmacy, Amrita Vishwa Vidyapeetham, AIMS Health Science Campus, Kochi 682041, Kerala, India; bijomathew@aims.amrita.edu; 4 Department of Pharmacy, and Research Institute of Life Pharmaceutical Sciences, Sunchon National University, Suncheon 57922, Korea

**Keywords:** hepatocellular carcinoma, immunotherapy, multidrug resistance

## Abstract

Hepatocellular carcinoma (HCC) has emerged as one of the most lethal cancers worldwide because of its high refractoriness and multi-drug resistance to existing chemotherapies, which leads to poor patient survival. Novel pharmacological strategies to tackle HCC are based on oral multi-kinase inhibitors like sorafenib; however, the clinical use of the drug is restricted due to the limited survival rate and significant side effects, suggesting the existence of a primary or/and acquired drug-resistance mechanism. Because of this hurdle, HCC patients are forced through incomplete therapy. Although multiple approaches have been employed in parallel to overcome multidrug resistance (MDR), the results are varying with insignificant outcomes. In the past decade, cancer immunotherapy has emerged as a breakthrough approach and has played a critical role in HCC treatment. The liver is the main immune organ of the lymphatic system. Researchers utilize immunotherapy because immune evasion is considered a major reason for rapid HCC progression. Moreover, the immune response can be augmented and sustained, thus preventing cancer relapse over the post-treatment period. In this review, we provide detailed insights into the immunotherapeutic approaches to combat MDR by focusing on HCC, together with challenges in clinical translation.

## 1. Introduction

Liver cancer is a major health concern and a leading cause of cancer-related death worldwide with an annual incidence of over 850,000 new cases globally [1]. Hepatocellular carcinoma (HCC) represents 90% of all primary liver cancers and principally occurs within the setting of chronic inflammation such as viral liver disease and alcoholic and non-alcoholic fatty liver diseases [2,3]. Forty to fifty percent of patients diagnosed with early stages of liver cancer are receptive to various curative approaches. Unfortunately, 70% of patients are diagnosed with disease recurrence within 5 years, and to date, no adjuvant therapies are available to forestall this complication [4]. Surgical procedures such as trans-arterial chemoembolization seem to be effective in the intermediate stage; however, such procedures are ineffective for advanced HCC stages. Patients suffering from advanced stages of HCC can benefit only through systemic therapies mainly with multi-kinase inhibitors. The sole multi-kinase inhibitor available for standard care for advanced HCC patients is sorafenib [5,6]. Sorafenib, however, has demonstrated very low survival benefits with limited rates of tumor response, suggesting the existence of primary and acquired drug resistance mechanisms [7,8]. Since the approval of Sorafenib, several clinical trials have shown a failure to achieve overall survival.

Several approaches have been deployed for years to sensitize resistant tumor cells by chemotherapy but with varying and limited positive results. In this context, the strong “immune-role” of tumor cells acquiring resistant phenotypes suggests that immunotherapeutic approaches can be novel strategies to target resistant tumor cells. Because of the unique anatomical organization, strategically designed tolerogenic environment, and a vast range of immune-cell depositories, the liver is the best target for immunotherapeutic approaches. A plethora of immunotherapy-based clinical studies is ongoing to combat this chemotherapy-resistant tumor. In this review, we aim to focus on the role of the liver in immune homeostasis and its deregulation in HCC together with various immunotherapeutic approaches to combat drug resistance and surveillance in HCC.

## 2. HCC Tumor Resistance: Signaling and Strategies

### 2.1. Drug Efflux Pump and Drug Metabolism

The complex signaling in tumor cells leads to decreased drug influx, increased drug efflux, alterations in molecular targets, intracellular drug metabolism, and enhanced repair of drug-mediated modifications, which further results in decreased pharmacological response and enhanced drug resistance in tumor cells. Prolonged usage of anticancer drugs may lead to the overexpression of drug efflux pumps, thus reducing their bioavailability at the tumor site. Several drug efflux pumps are activated during chemotherapy, which includes MDR1, also referred to as ABCB1 or P glycoprotein, MRP1 (ABCC1), MRP2 (ABCC2), MRP3 (ABCC3), and ABCG2 [9,10]. Hence, recent therapeutic anticancer strategies involve efflux pump antagonists against resistant cancer cells. Additionally, OATP1B1 and OATP1B3 drug influx pumps, which are downregulated in tumor cells, lead to decreased drug intake [11,12] (Table 1).

### 2.2. Alteration of DNA Repair Pathways and Chemoresistance in HCC

The development of hepatocarcinogenesis within a chronic inflammatory milieu results in a cascade of genetic alterations and genomic instability. Dysregulation of DNA damage repair may lead to the transmission of mutations to next-generation cells, culminating in cancer initiation. Upregulation of DNA repair enzymes is a major hallmark of resistant tumor cells, which rapidly repair DNA damage, conferring resistance to DNA damaging chemotherapeutic agents.

Ras-induced upregulation of ERCC-1—a key enzyme involved in nucleotide excision repair (NER)—protects HCC cells from platinum-based anticancer agents [32]. Interestingly, Flap endonuclease-1 (FEN-1) is a key enzyme involved in base excision repair (BER), which additionally possesses gap endonuclease (GEN) activity, which is involved in the induction of apoptosis. A study on HCC revealed that lower doses of cisplatin activate the DNA repair capability of FEN-1, which leads to cisplatin resistance, whereas high doses of cisplatin activate the endonuclease activity of FEN-1, resulting in apoptotic death of tumor cells [33]. Checkpoint kinase 2 (Chk2) is a multifunctional enzyme that can induce cell-cycle arrest and apoptosis conferred by DNA damage. Long-term paclitaxel therapy leads to dysregulation of Chk2 levels, leading to the emergence of resistance in HCC [34]. Blockade of DNA damage sensor, ataxia telangiectasia mutated (ATM), interferes with Akt signaling and sensitizes HCC cells to Sorafenib [35]. Apurinic/apyrimidinic endonuclease (APE1) is a dual functioning protein that regulates DNA repair as well as redox activity of transcriptional factors. APE1 silencing suppressed radioresistance in HCC and improved cytotoxicity and apoptosis induction by irradiation in vivo and in vitro [36] (Table 2).

### 2.3. Tumor Microenvironment

The tumor microenvironment (TME) is an important factor involved in the development of resistance in HCC cells. TME comprises both cellular and non-cellular components. The cellular components include hepatic stellate cells, fibroblasts, immune cells (T_reg_ cells, T_h_ cells, and T_c_ cells), and tumor-associated macrophages [47]. The non-cellular components include extracellular matrix proteins, pro-inflammatory and anti-inflammatory cytokines, proteolytic enzymes, and growth factors.

The overexpressed cancer-associated fibroblasts (CAFs) in HCC can alter the transcriptome features of HCC cells, thereby accelerating the migration, proliferation, and invasion of cancer cells. In addition, the ability of CAFs to activate non-cellular components such as pro-inflammatory and anti-inflammatory cytokines can be correlated with their role in the invasion, proliferation, and angiogenesis of cancer cells. In contrast, CAFs also augment the production of growth factors such as epidermal growth factor (EGF), hepatocyte growth factor, fibroblast growth factor (FGF), and Wnt family receptors. Therefore, CAF-mediated activation of multiple signaling pathways results in the emergence of chemoresistance in HCC.

Impairment of immune responses mediated by CD8+ T cells and NK cells are also involved in the development of chemo-resistance in HCC [48,49]. Activation of matrix metalloproteinases (MMPs) is also critical in the neoplastic transformation of cells via the downstream PI3K/PTEN/AKT/mTOR pathways [50], and different types of MMPs—such as MMP-2 and MMP-9—play a crucial role in tumor proliferation via the cleavage of the prominent components of the extracellular matrix, consequently prompting cancer-cell invasion [51]; this process hijacks apoptosis signaling in cancer-causing inflammation, tissue remodeling, tumor cell growth, and HCC metastasis [52]. Activation of hepatic stellate cells during liver injury triggers the release of type I collagen, which promotes the epithelial–mesenchymal transition (EMT) of hepatic cells [53] (Table 3 and Table 4).

### 2.4. Micro RNA (miRNA) in HCC

miRNAs are a class of recently identified cancer hallmarks involved in the neoplastic transformation as well as desensitization of tumor cells to chemotherapeutic drugs. These highly conserved short (~20 nucleotides) non-coding RNA molecules are involved in mRNA silencing as well as in post-transcriptional modifications of genes associated with cell proliferation and chemoresistance [65,66,67].

Altered miRNA expression in cells occurs because of various molecular disarrays such as epigenetic modifications, gene sequence alterations, deregulated transcription factors, and biased miRNA biogenesis and processing [68,69,70]. Dysregulated miRNAs in correlation with their regulatory networks of mRNAs, proteins, and other non-coding mRNAs alter the genes of cell apoptosis, cell proliferation, autophagy, drug efflux, and EMT, giving rise to a resistant malignant transformation of cells [71,72].

A considerable amount of evidence suggests an oncogenic role of miRNA in HCC. Long-term treatment with sorafenib, cisplatin, and doxorubicin may lead to the generation of impaired miRNAs in the body and elicit resistance against chemotherapy. Doxorubicin can downregulate miRNAs such as miR-122, miR-101, miR-26a/b, miR-375, miR-223, and miR520b and their gene targets *ABCB1*, *ABCF2*, *PKM2*, *Mcl-1*, *ULK1*, *YAP1*, *AEG-1*, *ABCB1*, and *ATG7*, respectively [73,74,75]. Sorafenib can upregulate miR-216a/217, miR-222, and miR-21 and targets their corresponding genes: *PTEN*, *SMAD7*, and *AKT* [76,77,78,79,80] (Table 5).

### 2.5. Chemoresistance due to Epigenetic Regulation

DNA methylation plays a key role in the development of epigenetic regulation in cancer that can generate chemo-resistance in HCC. During chemotherapy, activation of hypermethylated genes such as *RASSFIA*, *APC*, *FZD7*, and *CDKN2A* promotes abnormal methylation in DNA and causes poor prognosis due to the development of chemoresistance [91]. Other reports suggest that 5-FU mediated transcriptional repression of miR-193a-3p promotes hypermethylation of DNA and the emergence of resistance. Hence, the suppression of DNA methylation is crucial for successful 5-FU therapy. However, high levels of tri-methylated histone H3 lysine 4 (*H3K4me3*)—a transcriptional suppressive gene—were found to be associated with poor survival, prognosis, and aggressive tumor features in HCC [92].

### 2.6. Topoisomerases in Chemoresistance

DNA topoisomerases are necessary enzymes that are critical for the maintenance of DNA duplexes. Higher expression of topoisomerase 2A (TOP2A) has been reported in numerous types of malignancies and is suggested to be a valuable prognostic marker for tumor progression, recurrence, and poor survival [93]. The levels of TOP2A were found to be elevated during long-term therapy with doxorubicin and contributed to chemoresistance development. The combination of a novel topoisomerase I inhibitor, tirapazamine (TPZ), with DNA damaging agents exhibited synergistic cytotoxicity and induced significant apoptosis in several HCC cell types [94]. Thus, targeting topoisomerases can be an appropriate strategy for HCC along with conventional therapy.

### 2.7. Cancer Stem Cells in Chemoresistance

The stem-cell model of cancer suggests that among cancerous cells, a subset of the cell population acquires stem cell-like properties, thus conferring the unique ability to differentiate continually and sustaining malignancy [95,96]. In the case of HCC, cancer stem cell (CSC) markers include epithelial cell adhesion molecules (CD133, CD90, CD44, CD24, and CD13), which provide resistance as well as a metastatic phenotype to the malignant cells via the activation of the Akt and Bcl-2 survival pathways [97,98].

### 2.8. Telomerase and Chemo-Resistance

The telomerase enzyme is overexpressed in many cancers. It bestows anti-apoptotic and chemo-resistant properties to cancer cells. Low doses of cisplatin were found to activate telomerase activity in human HCC cells. Following this, it was observed that siRNA against human telomerase reverse transcriptase (hTERT) and cisplatin therapy could act synergistically to suppress HCC progression compared to monotherapy [99]. Thus, upregulation of hTERT expression by cisplatin depends on NF-κB, which contributes to chemotherapy resistance in HCC cells [100].

### 2.9. Impaired Lipid Metabolism

Altered lipid biosynthesis and metabolism play a key role in cancer pathogenesis. Stearoyl-CoA desaturase (SCD), an enzyme that regulates lipid homeostasis in the liver, is overexpressed in HCC. SCD downregulation by pharmacological or genetic means may lead to increased sensitivity toward chemotherapy-induced cell death. The administration of 5-FU elevates SCD levels through the PI3K and JNK pathways in a time-dependent manner [101]. Another enzyme involved in lipid metabolism is carbonyl reductase 1 (CBR1), which protects the cells from lipid peroxidation. CBR1 accelerates the action of angiogenesis promoter, HIF-1α, a transcription regulator, leading to chemoresistance in HCC [102].

## 3. Immunotherapy: A Novel Weapon against HCC

Cancer treatment primarily relies on chemotherapy, radiotherapy, and surgery. Although several signaling-targeted drugs have been rapidly developed, the cure for cancer remains elusive. Immunotherapy is gaining considerable attention as a new generation strategy to combat chemo-resistant HCC because liver cancer is mostly an immunological tumor. In this review, we discuss the anatomical and physiological features of the liver that make it suitable for immunotherapy and the various approved, as well as ongoing, immunotherapy strategies to combat resistant HCC.

### 3.1. Immune Contexture of HCC

#### 3.1.1. Immunological Organization and Immune Regulation of the Liver

The liver is an organ of synthesis, storage, and metabolism. Because of the ideal positioning of the liver in the abdominal cavity, beneath the diaphragm, and on the top of the stomach, right kidney, and intestine, the liver is continuously exposed to nutrients, microbe/pathogen-derived molecules, and toxicants [103]. The characteristic hemodynamic pattern of the liver allows it to receive both arterial and venous blood through the hepatic artery and portal vein, respectively. The portal venous circulation supplies 80% of the blood to the liver from the spleen, pancreas, and GI circulation, whereas the remaining 20% is supplied by the hepatic artery [104,105]. As the blood flows through thousands of capillary-like microstructures in the liver called “liver sinusoids”, blood flow slows down. The decelerated blood flow and pressure drop allow the maximal exchange of metabolites as well as optimal interaction of pathogen-derived molecules (rich in portal blood) with diverse immune cells in the liver [106].

**Figure 1:**
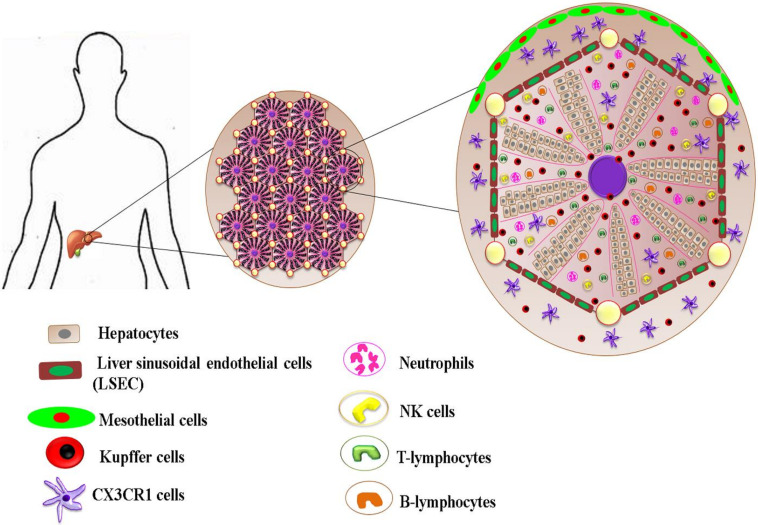
Anatomical organization and distribution of various immune cells in the liver. The sinusoidal endothelial cells of the liver in the sinusoidal lining directly interact with resident liver macrophages, KCs. The liver harbors varying proportions of lymphoid cells such as natural killer cells, natural killer T cells, γδT cells, and liver transiting or resident T cells.

Liver sinusoidal endothelial cells (LSECs) form a specialized sinusoidal lining. LSECs closely monitor portal circulation and uptake, degrade circulating molecules, and directly interact with resident liver macrophages, Kupffer cells (KC), which constitute the major macrophage depository of the body [107,108]. In addition, the liver harbors a varying proportion of lymphoid cells such as natural killer cells, natural killer T cells, gamma delta T cells (γδT cells), and liver transiting or resident T cells, which are significantly higher than in peripheral blood [109]. This unique anatomical organization leads to continuous exposure to gut-borne pathogens as well as foreign non-pathogenic molecules that create an immunological load within the liver. Thus, the liver strictly maintains immunotolerance against gut-derived molecules, whereas the immune response provides immunity against threats [110]. All these features are strategically designed to make the liver an immune sentinel, i.e., a guard for balanced immune regulation (Figure 1).

In the basal condition, the liver promotes a tolerogenic environment because of the complex interaction between hepatic cellular components such as KCs, LSECs, dendritic cells (DCs), hepatocytes, and peripheral leukocytes. LSECs control the immune response by overexpressing inhibitory molecules such as program death receptor ligand-1(PD-L1) while suppressing co-stimulatory molecules such as CD80, thereby inducing CD4+ and CD8+ T cell tolerance [111,112]. In addition, anti-inflammatory cytokines such as TGF-β and IL-10 downregulate MHC class I and II molecules in LSECs, thus limiting their antigen-presenting functions [113,114]. LSECs also inhibit dendritic cell-mediated T-cell activation [94]. KCs also follow the same mechanism of tolerance induction through the suppression of MHC molecules, activation of inhibitory cytokines such as IL-10, TGF-β, and prostaglandins (PG), and expansion of inhibitory regulatory T cells (T_reg_) cells [115,116]. Pattern recognition receptors in hepatocytes recognize and degrade microbial-associated molecular patterns and damage-associated molecular patterns in the portal circulation without producing pro-inflammatory cytokines, thus preventing abrupt immune activation.

Although the liver maintains a tolerogenic milieu, it can build a rapid and appropriate immune response against pathogens and tumor cells. Upon active liver injury, activated KCs produce inflammatory cytokines such as TNF-α, IL-6, and IL-1. In addition, CD141^+^ myeloid DCs induce T-cell–mediated production of IFN-γ and IL-7 [117]. This altered inflammatory environment activates T cells to function as effector T cells and clear pathogens. As discussed above, the liver functions in a strictly regulated immunological milieu and alterations in this immunological network lead to liver diseases, including HCC.

#### 3.1.2. Immune Responses in HCC

##### Cancer-Immunity Cycle

When normal cells undergo neoplastic transformation inside the body, our immune system designs an immune cycle to eliminate cancerous cells. Antigen-presenting cells (APCs) capture the tumor-associated antigens (TAAs) released from tumor cells because of necrosis on their surfaces through the major histocompatibility complex (MHC). The binding of TAA-linked APCs to the specific T-cell receptors (TCR) of CD8+ T cells leads to the activation of CD8+ T cells to the effector T-cell phenotype and cytotoxic T lymphocytes (CTL). Simultaneous binding of APC to CD4+ T cells also stimulates the activation of CD8+ T cells. The CTLs infiltrate the tumor microenvironment and destroy cancer cells by binding with MHC-presented antigens with TCR. Eliminated cancer cells further release antigens that reactivate the next immune cycle [118,119]. Besides the antigen presentation to CD4/CD8+ T cells, many other signaling molecules are required for efficient T-cell activation, which includes activation of co-stimulatory molecules such as CD28-CD80/86, CD37-CD137L, and CD27-CD70 and suppression of co-inhibitory molecules such as CTL associated protein 4(CTLA-4), programmed cell death protein 1(PD-1), killer immunoglobulin-like receptor-MHC class I and II molecules, and T-cell immunoglobulin domain and mucin domain3. Together with this, pro-inflammatory cytokines such as IL-1 and IFN- are essential to sustain T-cell priming [120]. During chronic infection/liver disease, the deregulated immune system exacerbates immunotolerance, which leads to further disease progression and tumor induction [121]. This indicates that cancer cells use their machinery to edit the cancer-immunity cycle and survive.

##### Mechanism of Immune Evasion in HCC

The chronically inflamed liver microenvironment possesses a higher amount of immune inhibitory cells such as T_reg_ cells and myeloid-derived suppressor cells and a lower amount of co-stimulatory molecules such as CD80 and CD86 [122]. HCC involves small amounts of CD4+ and CD8+ effector T cell expressing prominent markers for T-cell exhaustion like layilin [123]. The abundance of T_reg_ cells in HBV+ patients is linked with the disease progression of HCC. In NAFLD and NASH, inflammation-induced cytotoxic CD8+ T cell exhaustion by IgA+ cells has been identified as a tumor-promoting mechanism [124]. Increased secretion of immunosuppressive cytokines such as IL-4, IL-5, IL-8, IL-10, and TGF-β together with the suppression of pro-inflammatory cytokines like IL-1, TNF, and IFN-γ also prevents T-cell priming [125]. In established HCC, in addition to T-cell exhaustion and the subsequent inhibition of Ag presentation, tumor cells create immunotolerance by checkpoint inhibition. The co-inhibitory receptor with its ligand is collectively called a checkpoint. Immune checkpoints confer protection from an excessive immune response. However, in malignancies, checkpoints are over-activated to escape immune clearance. The checkpoint formed by CTLA-4 and PD-1 is prominently found in HCC. CTLA-4 on T cells and T_reg_ cells inhibit T-cell activation by: (1) preventing binding of CD80 and CD86 on their co-stimulatory receptor CD28; (2) removal of CD80 and CD86; and (3) increasing the inhibitory effect of T_reg_ cells. PD-1 binds its ligands, PD-L1 and PD-L2, thus inhibiting the functioning of CTLs via: (1) increased apoptotic clearance of CTLs; (2) preventing CTL proliferation; and (3) reduced phosphorylation of TCR [126,127].

### 3.2. HCC as an Ideal Candidate for Immunotherapy

Unlike other malignancies, HCC is a complex pathology that arises from a chronic inflammatory environment, usually diagnosed at an advanced stage with a highly resistant phenotype. Most treatment modalities for HCC, including conventional chemotherapy and novel targeted therapies, fail to improve the overall survival of patients. Because the aggravated immunotolerance in HCC promotes immune evasion and survival of tumor cells, immunotherapy approaches to stimulate the immune system against cancer cells seem to be ideal for HCC.

#### 3.2.1. Adoptive Cell Therapy

Adoptive cell therapy (ACT) is laying the foundation of immunotherapeutic approaches, which involve programming the patient’s lymphocytes to destroy tumor cells. ACT is performed by loading tumor antigens or cytokines on isolated lymphocytes followed by ex-vivo expansion and reinfusion to the patient [128]. ACT comprises four subtypes: cytokine-induced killer (CIK) cells, tumor-infiltrating lymphocytes (TIL), natural killer (NK) cells, and chimeric antigen receptor (CAR) T cells.

➢ Cytokine-induced killer-cell therapy

CIK cells are separated from peripheral mononuclear cells and are cultivated ex vivo with an array of pro-inflammatory cytokines such as IL-1, IL-12, and IFN-γ to prime them to an effective cytotoxic phenotype. Numerous CIK cell therapies have been effectively examined in various HCC studies [129]. In a phase III trial, Lee et al. demonstrated the effect of CIK as adjuvant therapy after surgical resection/radiofrequency ablation in 230 patients with HCC which resulted in augmented recurrence-free overall survival [130]. In another study, Yu et al. used CIK cell therapy as the primary treatment for 132 patients and demonstrated overall and progression-free survival [131]. These data indicate that immunotherapy is effective in improving survival rates as well as reducing recurrence in HCC patients when given as monotherapy or as adjuvant therapy.

➢ Tumor-infiltrating lymphocyte therapy

TILs are isolated from surgically resected tumors and grown in a culture medium enriched with IL-2 and CD-3 agonists to stimulate T-cell proliferation. These primed T cells with cancer-specific immunity are reinfused into the patients [132,133]. The application of TIL therapy has been limited to a few carcinomas such as melanoma because the process of culturing and purification of TILs is laborious.

➢ Natural killer cell therapy

NK cells are the major players of the innate immune system. They are capable of eliciting an immune response against viruses and tumor cells without any prior sensitization. This makes NK cell therapies an attractive candidate for the management of solid tumors such as HCCs [134]. In a cell-line–based study, NK cells were resected from healthy volunteers after stimulation with a hepatoma cell. These expanded and activated NK cells exhibited significant cytotoxicity in various HCC cell lines and showed a remarkable additive effect upon the combination with the sole drug, sorafenib [135]. Several clinical studies have utilized NK cells in different types of cancers. In one such study, 10 HCC patients with cirrhosis underwent liver transplantation followed by an adoptive immune cell therapy using liver NK cells derived from a cadaveric donor liver, which was found effective as it increased cytotoxicity and prevented recurrence with no treatment-related adverse events [136]. Nowadays, genetically modified NK cells are being increasingly accepted due to their targeted or site-specific actions. Similar to the concept of CAR-T cells (discussed below), CAR-NK cells have been developed, which exhibit extended cytotoxicity with minimal risk of autoimmune responses. Furthermore, CAR-NK cells are superior to CAR-T cells due to their well-controlled release of cytokines [137,138]. Currently, CAR-NK cells are under active clinical trials to confirm their safety and efficacy.

➢ Dendritic cell therapy

Dendritic cells are the major APCs of our immune system that capture, process, and present antigens to T cells, thereby inducing T-cell mediated immunity. Tumor cells edit or suppress DC proliferation and its ability of antigen presentation; thus, DC-mediated immune response is restricted in tumor cells [139]. In ACT, IL-4 and granulocyte-macrophage colony-stimulating factor (GM-CSF) mediated ex-vivo DC stimulation, and further reinfusion to the patients enabled the activation of the host immune response. Nowadays, TAA or TAA-derived peptides are also being used as antigens to prime DCs to mature DCs with potent antigen-presenting activity [140]. To avoid abrupt immune activation resulting from the administration of TAA or TAA-derived peptides, DC vaccines have been developed by fusing DCs and tumor lysates or by transducing DNA or RNA sequences encoding for TAA. Ilixadencel, an IL-4 and GM-CSF stimulated DC injection, was studied as monotherapy and as a combination with Sorafenib in the phase 1 clinical trial. The study showed an increased cytotoxic T-cell population with the occurrence of only one grade 3 adverse reaction [141].

#### 3.2.2. Genetically Modified T-Cell Therapy

Transduction of T cells with TAA-specific TCR or CAR creates tumor antigen-specific T cells with potent cytotoxicity.

➢ TCR-engineered T-cell therapy

**Figure 2:**
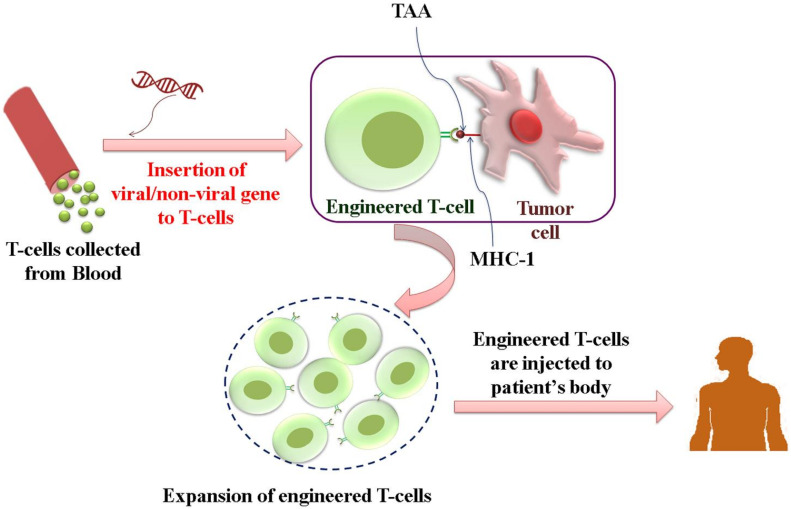
Schematic representation of engineering TCR to target HCC tumor-specific antigens. T lymphocytes are isolated from the blood and genetically modified to express T-cell receptors, which can recognize a specific tumor-associated antigen and elicit cell-specific cytotoxicity. After expansion, these engineered T cells are injected into the patient.

APC captures tumor antigens and presents it to the T-cell receptor (TCR), which is made of α and β chains linked to CD3 on the surface of T cells, thus activating T cells to the effector phenotype. Antigen-specific T-cell immune responses can be achieved by modifying TCR to express tumor antigen-specificα and β chains and thereby programming T cells to recognize HCC-specific antigens such as AFP, hTERT, MAGE, and NY-ESO-1 [142,143,144,145]. In a pre-clinical study, genetically modified TCR to recognize HCV and AFP antigens were developed, and HepG2 cells with target antigens were grown in an immune-deficient mouse model. This modified TCR-T cell therapy demonstrated an antigen-specific immune response in-vivo. However, future clinical trials are essential for its clinical translation [146]. A phase 1 clinical trial with AFP targeting TCR-T cells involving patients with advanced HCC is currently ongoing (NCT03132792) (Figure 2).

➢ CAR-T cell therapy

**Figure 3:**
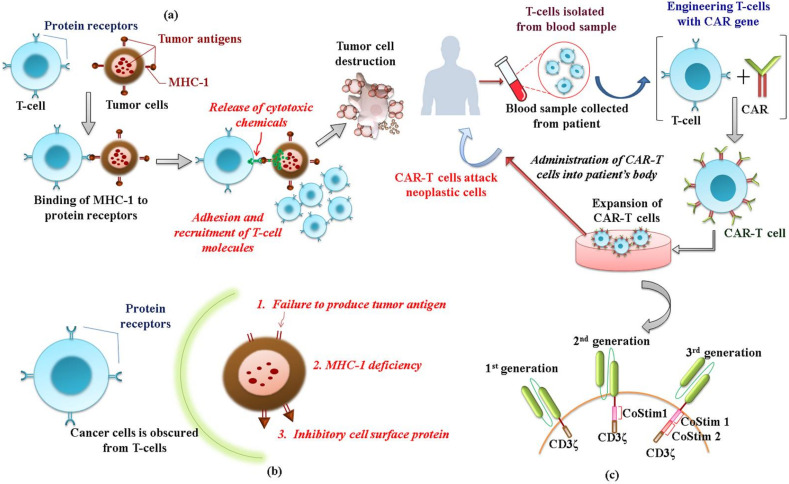
(**a**) Active immune surveillance against tumor cells. APCs capture TAAs released from tumor cells because of necrosis to its surface through the MHC. The binding of TAA-linked APC to the specific TCR of CD8+ T cells leads to its activation of the effector T-cell phenotype and CTL. (**b**) Immune evasion strategies of tumor cells. Tumor cells escape from T cell-mediated anticancer immunosurveillance mainly via the inhibition of production and presentation of TAA, deficient expression of MHC-1, and expression of immune inhibitory surface proteins. (**c**) CAR-T cell therapy. T cells isolated from the patient’s blood were transduced with a CAR gene for modified antigen receptors that can specifically target a tumor-specific antigen and destroy it without the need of MHCs (unlike T cells) and immunization (unlike vaccine). The expanded and screened CART-T cells are reinjected into the patient. Structurally, CAR is made of an extracellular antigen-binding domain of a tumor-specific antibody connected to an intracellular domain comprising CD3 of TCR via a transmembrane hinge region. Structural modifications of the intracellular domain with a costimulatory domain of CD28 results in the formation of second-generation CARs, whereas third-generation CARs comprise two co-stimulatory domains.

CAR-T cells are T cells that carry an additional gene with a modified antigen receptor that can specifically target a tumor-specific antigen and destroy it without requiring MHCs (unlike T cells) and immunization (unlike vaccine). Structurally, CAR is made of an extracellular antigen-binding domain of a tumor-specific antibody which is connected to an intracellular domain comprising CD3 of TCR via a transmembrane hinge region. Structural modifications of the intracellular domain with a costimulatory domain of CD28 result in second-generation CARs, whereas third-generation CARs are made of two co-stimulatory domains. CAR-T cell therapy is performed by viral-mediated CAR gene delivery to the T cells collected from the blood samples of patients, followed by colony expansion and reinfusion to the patients. Recently, fourth-generation CAR-T cells were developed with the ability to release pro-inflammatory cytokines such as IL-2 to prime T-cell activation [147] (Figure 3). This rapidly emerging approach is currently gaining acceptance as it is superior to modified TCR therapy and vaccines due to its proven efficacy in clinical trials of lymphoma.

Glypican-3 (GPC-3) is a tumor-associated antigen that is prominently expressed in 90% hepatocarcinoma cells with restricted or low expression in normal cells [148]. In a pre-clinical study with an orthotopic Huh-7 xenograft model, the engineered third-generation CAR-T cells targeting GPC-3 demonstrated a significant cytotoxic effect in GPC-3 positive cells with minimal off-target effects. In another study, T cells with two CARs co-expressing GPC-3 and asialoglycoprotein receptor-1 (ASGR1) were engineered. This dual-targeted CAR-T cell showed a specific and potent cytotoxic immune response in GPC3 + ASGR1 + HCC tumor xenografts with no off-target and minimal on-target toxicity. Although CAR-T cell therapy has proved efficacious in cancers such as acute lymphoblastic leukemia (ALL), it is still in the state of infancy in the case of HCC [149]. More than six clinical trials are currently ongoing for HCC with different antigens targeting CAR-T cells.

#### 3.2.3. Immune Checkpoint Inhibitors (ICI)

The cancer immunity cycle is balanced to elicit adequate antitumor immune activation while simultaneously avoiding abrupt immune activation via co-stimulatory and co-inhibitory molecules. However, cancer cells have developed strategies to escape immune surveillance. One such strategy is by the overexpression of co-inhibitory molecules which, along with their corresponding ligands, collectively form an immune checkpoint.

Immune checkpoint cascades are highly overexpressed in all liver-related complications such as cirrhosis, viral hepatitis, and HCC. Checkpoint targets are also involved in the resistance to sorafenib because its targets of inhibition include various apoptotic-immune signal transducers. Currently, checkpoint inhibition strategies are considered as an emerging tool for the treatment of HCC, a method that is also utilized as adjuvant therapy for cancer. Moreover, immune checkpoint inhibitors can prevent the relapse of cancer and hepatitis virus [150,151].

Immune checkpoints such as cytotoxic T lymphocyte-associated antigen-4 (CTLA-4), PD-1, PD-L1, V-domain Ig suppressor of T cell activation (VISTA), T cell immunoglobulin and mucin domain containing-3 (TIM-3), lymphocyte-activation gene-3 (LAG-3), and OX40 are reported to be prominently activated in HCC cells and involved in its prognosis. The clinically prominent immune checkpoint molecules are CTLA-4 and PD-1/PD L1 [152,153]. Overexpression of these molecules during cancer progression downregulates the immune response by suppressing cytotoxic T cell activation.

**Figure 4:**
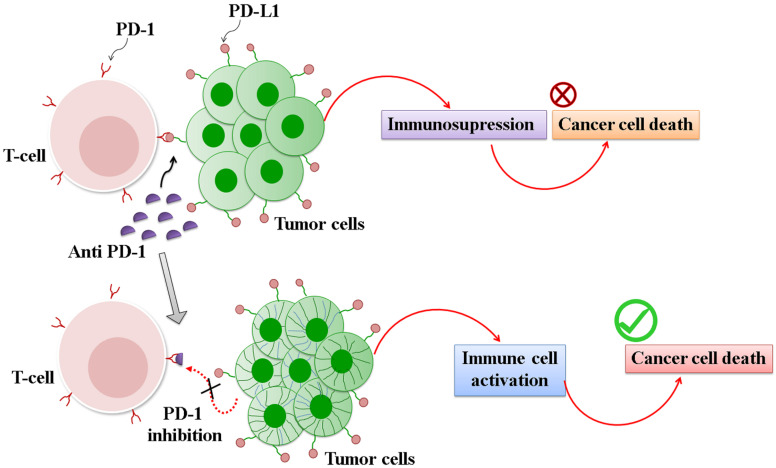
Mechanism of action of PD-I immune checkpoint inhibitors. PD-1 is a protein present on the T-cell surface. The corresponding ligand of PD-1 is called PD-L1, which is overexpressed in cancer cells. The interaction PD-1 and PD-L1blocks the cytotoxic effect of T cells. Pembrolizumab, a PD-1 inhibitor, prevents the interaction of PD-1 with its corresponding ligand and elicits an immune reaction.

Several studies have reported that PD-L1 overexpression induces resistance in HCC during sorafenib therapy [154]. According to a study conducted by Liu et al., DNA methyltransferases (DNMTs) and PD-L1 are the key factors that lead to sorafenib resistance in HCC. Considering immunotherapy is a rising treatment modality for cancer, PD-1/PD-L1 can be a potent target for immunotherapy [155]. Nivolumab (NCT01658878), marketed as Opdivo, is the first FDA-approved checkpoint inhibitor for HCC. Opdivo is a human monoclonal antibody that blocks the interaction among PD-1, PD-L1, and PD-L2 and upregulates the cytotoxic T cells for immune reaction. The binding of these ligands to the PD-1 receptor harbored on T cells inhibits T-cell proliferation and cytokine production, which are responsible for the immune reaction [156]. The upregulation of the PD-1 ligands occurs in most tumors, and signaling through this pathway can inhibit active T-cell immune surveillance of tumors [157]. Thus, nivolumab blocks checkpoint-mediated immune evasion of tumor cells and facilitate immune clearance [158]. Alternatively, pembrolizumab (NCT02702414) is a PD-1 antibody that is currently under Phase II clinical trials [159]. A dose of 200 mg of Pembrolizumab was administered at intervals of 3 weeks to sorafenib-intolerant patients, who were considered as cohort 1 in the study, and patients without a history of previous systemic treatment were considered as cohort II study. In this study, 104 sorafenib-intolerant patients were reported at a meeting of the American Society of Clinical Oncology (ASCO) in 2018, with promising results as a second-line treatment [160]. Furthermore, in a phase II trial conducted at a single institute at the University of Miami, the results obtained were approximately similar to those obtained in sorafenib-refractory or sorafenib-intolerant patients. Pidilizumab, formerly known as CT-011, another humanized monoclonal antibody, which can bind with the PD-1 immune checkpoint molecule, is under clinical trials for the treatment of cancer and other infectious diseases [161,162]. However, current studies suggest that the prime target for pidilizumab is the Delta-like 1 (DLL1) receptor, and its binding to PD-1 is only secondary. At present, various reports state that inhibition of PD-L1 and DNA methyl transferase-1 (DNMT1) significantly prevents the growth of Sorafenib resistant HCC cells in-vitro, and, thus, PD-1 and PD-L1 are considered novel valuable treatment options for Sorafenib resistant HCC [163] (Figure 4).

**Figure 5:**
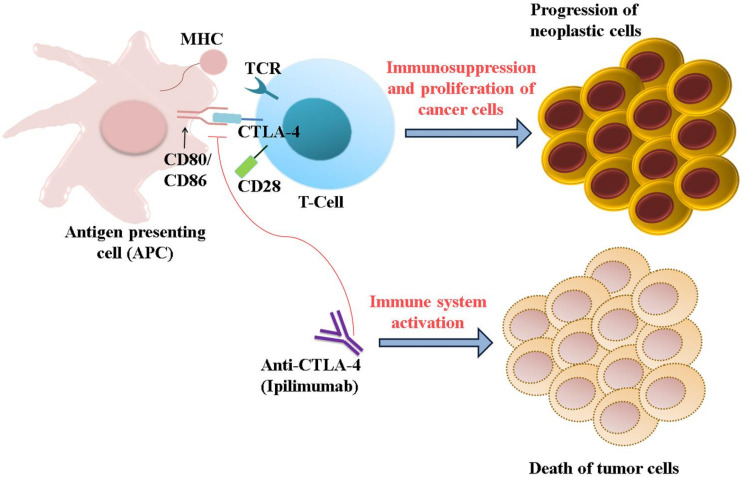
Mechanism of action of CTLA-4 immune checkpoint inhibitor. CTLA-4 can bind with the CD80/CD86 proteins, which are overexpressed in APC. This interaction blocks the cytotoxic effect of T cells by blocking the binding of T cells with the MHC protein present in cancer cells. Ipilimumab, an anti-CTLA-4 antibody, forestalls the interaction between CD80/CD86 and CTLA-4 and elicits an immune response.

In the case of CTLA-4–mediated checkpoint inhibition therapy, monoclonal antibodies such as ipilimumab and Tremelimumab are used for the treatment of HCC and hepatitis. The partial response rate of Tremelimumab was found to be 17.6%, the disease control rate was 76.4%, and the time taken for progression was 6.48 months. Moreover, viral loads of HCC were significantly decreased, and no patients experienced any immune-related adverse events (irAEs) or hepatotoxicity. The aforementioned studies demonstrate that tremelimumab is a safe anti-tumor and anti-viral drug for hepatitis C-induced HCC [164] (Figure 5).

TIM-3 is another potent target for checkpoint inhibition therapy. TIM-3 is generally overexpressed on the surface of CD4+ helper T cells and CD8+ cytotoxic T cells [165]. TIM-3 protein upregulation leads to cytotoxic T-cell inactivation and suppresses the immune clearance of tumor cells [166]. Thus, this protein plays an important role in the immunosurveillance in cancer progression. A dual blockade antibody (NCT03680508), which can block both PD-1 and TIM-3, is currently under phase II trials [167] (Table 6).

#### 3.2.4. Vaccines

Cancer vaccines represent an active immunotherapeutic approach that primes the immune system against tumor cells by delivering specific tumor-associated antigens. Because DCs constitute a major portion of hepatic APCs, they are largely employed for vaccine development. In general, three strategies are applicable for vaccine development: (i) pulsed DC vaccine (as discussed earlier); (ii) peptide-based vaccine; and (iii) DNA-based vaccine. In a phase II clinical study, 41 patients received GPC-3 peptide vaccine one year after curative treatment (surgery or radiofrequency ablation (RFA)). The study population demonstrated a significant decrease in recurrence rate as compared with a group that received only surgery [168]. In a prospective phase I trial, patients with advanced HCC received a subcutaneous injection of AFP-derived peptide. During the study period, one patient exhibited a complete response, defined as “tumor regression” without new lesions, and eight patients exhibited decreased tumor growth without the occurrence of adverse events. Similarly, in another study, two patients with AFP+ tumor treated with an AFP-DNA vaccine followed by an adenovirus immune-boosting exhibited AFP-specific CD4+ and CD8+ T-cell–mediated cytotoxic response [169].

Although a wide range of TAAs is linked to HCC development, only a few of them such as AFP, GPC-3, and MRP-3 have demonstrated promising clinical outcomes in vaccine development. Therefore, only a limited number of vaccine-based clinical trials have been conducted against HCC. In this context, project Hepavac, which started in September 2013, is important. The main objective of this project is the identification of multiple HCC-specific TAAs and the development of a multi-targeted, multi-peptide vaccine for HCC together with personalized treatment approaches. Hepavac-101 is a significant milestone from project Hepavac and is recognized as the first-in-man cancer vaccine targeting 16 new and overexpressed TAAs [170].

#### 3.2.5. Oncolytic Viruses

These are a novel immunotherapeutic approach in which viruses are designed to specifically target and multiply within tumor cells. At the end of the lytic cycle, tumor-invaded viruses cause oncolysis with the release of new virions that further destroy the remaining cancer cells and additionally activate the host immune response [171]. The oncolytic viral vaccine, Pexa-vec, is currently under a phase III clinical trial and is being evaluated as monotherapy or as a combination therapy with sorafenib [172]. Poxvirus has been evaluated in 30 HCC patients in a phase II clinical trial and demonstrated a dose-dependent improvement in patient survival.

### 3.3. Combination Strategies for Immunotherapy for HCC

The combination of immunotherapy with conventional chemo-surgical-locoregional therapies is gaining acceptance as a promising treatment strategy to activate anti-cancer immunity as well as to improve the overall survival of patients. Different combination strategies have been reported in preclinical as well as in Phase I and II clinical trials. Angiogenesis/neovascularization is a major cancer hallmark, and the vascular endothelial growth factor-A (VEGF-A), which is produced in the tumor cells by tumor-associated macrophages and fibroblasts, plays an important role as an angiogenesis-inducer. Increased VEGF-A expression could directly promote T_reg_-cell proliferation and recruitment. In addition, VEGF-A has an indirect link with the functioning of T cells. Fas-ligand-expressed tumor endothelial cells suppress cytotoxic CD8+ T lymphocytes and activate T_reg_ cells. VEGF-A activates Fas-ligand expression, thereby indirectly mediating cytotoxic T-cell (T_c_) exhaustion. Besides the role in angiogenesis, the immunomodulatory role of VEGF-A in various cancers including HCC makes VEGF inhibitors an ideal combinatorial candidate with ICI in immunotherapy. In a phase III clinical trial called IMBRAVE 150, anti-PD-L1 mAb, atezolizumab, is combined with anti-VEGF, bevacizumab. The anti-angiogenic, as well as the immunomodulatory effect of bevacizumab, improved the effect of atezolizumab, and a better additive effect superior to that of the multi-kinase inhibitor sorafenib was obtained. Similarly, a combinatorial approach using lenvatinib and pembrolizumab was evaluated in a phase IB study and demonstrated a considerable cytotoxic effect. Due to this promising result, the study was extended to the phase III level by comparing lenvatinib with the lenvatinib-pembrolizumab combination in advanced HCC patients [173].

Similarly, combination strategies using sorafenib and newer multi-kinase inhibitors for anti-PD-1therapy are currently being studied in more than three clinical trials (NCT03211416, NCT01658878, and NCT02988440) [174]. The combination of PD-1 inhibitors with the TGF-β1 receptor inhibitor, galunisertib, was evaluated in various pre-clinical models of HCC and resulted in the disruption of intra-tumor TGF-β signaling and the activation of anticancer immunity. Recently, a fusion protein was developed by combining monoclonal antibodies against PD-1 with the extracellular domain of the TGF-β1 receptor [175]. This dual immune-targeting fusion protein could counteract the immune evasion of tumor cells by boosting the innate and adaptive immune system.

ICI was also studied in combination with various locoregional therapies because it could improve the diversity of cytotoxic CD8+ T cells with the suppression of inhibitory T_reg_ cells. A pilot study of 32 HCC patients exhibited a dramatic increase in cytotoxic CD8+ T cells followed by systemic therapy of tremelimumab together with RFA. Following this result, similar strategies such as combination therapies of nivolumab with TACE and pembrolizumab and nivolumab with radioembolization are being evaluated in various clinical trials [176].

## 4. Challenges and Future Perspectives

Immunotherapy is a novel approach to treat HCC which aims to be more promising and effective than the conventional treatment methods. In contrast, cancer immunotherapies have their limitations during long-term treatment. In patients with HCC, infiltration of lymphocytes occurs in association with the treatment of checkpoint inhibitors, leading to the generation of immune-related adverse effects during and after the treatment. Thus, it is important to develop proper methods to identify such types of adverse drug reactions. Similarly, assessment of treatment responses is also critical [177]. In the field of immunotherapy, identifying clinically significant biomarkers is challenging. Antibody-based drugs are designed to detect tumor-specific antigens present in neoplastic cells (neo-antigens). However, these neo-antigens are present in both cancer and non-cancerous cells, and the non-specific binding of an immunotherapeutic drug may lead to the development of off-target effects in the body [178].

Only one biomarker is available for immunotherapy, which is termed cancer-testis antigen (CTA). CTAs have been broadly investigated and are considered a promising immunotherapeutic target. CTAs may also serve as optimal targets for cancer immunotherapy directed against CSCs. They are expressed by CSCs and play a role in CSC differentiation and tumor biology. Most cells comprising a tumor mass are thought to result from the differentiation and cloning of a small number of CSCs that maintain and constantly “feed” the growth of the tumor. With the evidence that CSCs exist in many different tumors, it is imperative to identify and understand tumor antigens expressed by CSCs [179,180].

Tumor heterogeneity, in contrast, sometimes impedes the efficacy of immunotherapy. During prolonged treatment, cancer cells can adopt certain survival mechanisms, and mutations are the major strategy adopted by cancer cells, which can lead to linear tumor progression in cancer cells. A high level of intra-tumor heterogeneity may also result in the development of resistance to therapy. Therefore, the effect of immunotherapy is limited in the case of cancer tissues possessing heterogeneity [181,182].

## 5. Conclusions

The definitive goal of cancer therapy is to effectively kill tumor cells with minimal side effects. Targeted therapies against cancer have been developing over the last several decades, and they have ultimately demonstrated improved efficacy over conventional chemotherapeutics. Unfortunately, numerous hurdles associated with current treatment strategies exist, and the “war on cancer” persists. Major obstacles such as failure of therapy and relapse due to the multi-drug resistance reduce the output of conventional therapies. Currently, there is an urge for novel cancer therapeutics and combination strategies. The future success of cancer treatment strategies will solely rely on whether these novel therapeutic strategies can efficiently and economically overcome the current treatment limitations. In this regard, immunotherapy is gaining increasing attention as the fifth pillar of cancer therapy because it has presented tremendous hopeful outcomes. The number of ongoing or approval-awaiting immunotherapy approaches in HCC bestows a light of hope to combat this life-threatening malignancy.

## Figures and Tables

**Table 1:** Involvement of efflux pumps in MDR and their inhibitors as chemosensitizers.

Transporter Involved in MDR	Effluxed Cytotoxic Drug	Drug Transporter Inhibitors as Chemosensitizers	
		Preclinical Reports	Clinical Reports
P-gp (ABCB1)	Doxorubicin, Daunorubicin, Etoposide, Teniposide, Methotrexate, Sorafenib, Paclitaxel, Vincristine, Vinblastine	Verapamil [13] Nifedipine [14] Nimodipine [15] Amiodarone [16] Cyclosporine A [17] Tacrolimus [18]	Quinine [19] Tesmilifene [20] Biricodar [21]
MRP1 (ABCC1)	Doxorubicin, Daunorubicin, Methotrexate, Irinotecan, Etoposide, Teniposide, Imatinib, Gefitinib, Vincristine, Vinblastine	Disulfiram [22] Pak-104P [23] Cyclosporin [24]	Biricodar [25] Quinine [19] Tacrolimus [26]
BCRP (ABCG2)	Doxorubicin, Daunorubicin, Epirubicin, Methotrexate, Irinotecan, Etoposide, Teniposide, Imatinib, Gefitinib.	Nifedipine [27] Dihydropyridine [28] Cyclosporine A [29] Ritonavir [30]	Elacridar [31]

**Table 2:** DNA damage response pathway in MDR and their inhibitors as chemosensitizers.

DNA Damage Response Mechanism	Affected Cytotoxic Drugs	Drugs Targeting DDR as Chemosensitizers	
		Preclinical Reports	Clinical Reports
Nucleotide Excision Repair	Cisplatin, Alkylating agents	F117 82 [37] UCN01 [38]	Ecteinascidin 743 [39]
Homologous Recombination	Doxorubicin	17-AAG [40] ImatinibErlotinib [41] PCI-24781 [42] B02 [43]	
Non-homologous end joining	Topoisomerase inhibitors	NU7441 [44]	
DNA mismatch repair	Cisplatin, Carboplatin	Decitabine [45]	
O6-methyl guanine DNA methyl transferase	Alkylating agents		O6-benzyl guanine [46]

**Table 3:** Altered tumor microenvironment and its modulation to combat drug resistance.

Tumor Microenvironment Associated Targets Involved in MDR	Drugs Modulating Tumor Microenvironment as Chemosensitizers	
	Preclinical Report	Clinical Report
COLI A2	Let-7 g [54,55]	
Extracellular matrix protein	PI 88 [56]	
VEGFR2, FGFR1		Brivatinib (NCT00858871)
VEGFR2, FGFR, PDGFR		TSU-68 (NCT00784290)
PDGFR		MEDI-575 (NCT01102400)
GPC3		GC33 (NCT01102400)

**Table 4:** Overexpressed cell survival pathways in MDR and their inhibitors as chemosensitizers.

Over Expressed Cell Survival Pathway	Cancer Type	Inhibitors of Cell Survival Pathways as Chemosensitizers	
		Preclinical reports	Clinical Reports
PI3k/AKT/mTOR	Ovarian, cervical, gastric, breast, colorectal, hepatocellular, thyroid, endometroid, glioblastoma, acute leukaemia	AZ D8055 [57] CC-2223 [58]	Rapamycin, RAD001 [59]
Hedgehog	Gastro oesophageal, pancreatic, hepatocellular, brain, non-small cell lung cancer, glioblastoma	arsenic trioxide [60] Itraconazole [61]	Sonidigib, buparlisib, vismodigib, saridigib, taladigib [62]
EGFR	Head and neck, breast, renal, cervix, esophageal, pancreatic, non-small cell lung, colon, liver, bladder, gastric		Ramucirumab [63,64] Erlotinib Lapatinib Cetuximab Vandetanib

**Table 5:** Micro RNA mediated regulation of drug resistance.

miRNA	Resistance Conferring Targets Modified by MiRNA
MiR-122	IGF-1R [81] SRF ADAM10 PDK4 SLC7A1 GALNT
miR-34a	BCL-2 [82]
MiR-27b	P53 [83]
let-7	Bcl-XL [84]
miR-193b	MCL1 [85]
miR-486	CITRON CLDN10 [86] AR
miR-367-3p	CITRON CLDN10 [87] AR
miR-338-3p	HIF-1α [88]
miR-142-3p	ATG5 ATG16L1 [89]
miR-7	TYRO3 TYRO3-AXL-MER [90]

**Table 6:** Immune checkpoint inhibitors for HCC treatment.

Inhibitor	Target	Reference/Trial ID
Nivolumab	PD L1	NCT01658878
Pembrolizumab	PD 1	NCT02702414
Pidilizumab	DLL 1, PD 1	NCT00966251
Ipilimumab	CTLA 4	NCT03510871
Tremelimumab	CTLA 4	NCT03638141
Dual blockade antibody	TIM 3	NCT03680508

## Data Availability

Not applicable.
